# The effect of calcium and magnesium on activity, immunogenicity, and efficacy of a recombinant N1/N2 neuraminidase vaccine

**DOI:** 10.1038/s41541-021-00310-x

**Published:** 2021-04-06

**Authors:** Luca T. Giurgea, Jae-Keun Park, Kathie-Anne Walters, Kelsey Scherler, Adriana Cervantes-Medina, Ashley Freeman, Luz Angela Rosas, John C. Kash, Jeffery K. Taubenberger, Matthew J. Memoli

**Affiliations:** 1 LID Clinical Studies Unit, Laboratory of Infectious Diseases, Division of Intramural Research, National Institute of Allergy and Infectious Diseases, National Institutes of Health, Bethesda, MD USA; 2 Viral Pathogenesis and Evolution Section, Laboratory of Infectious Diseases, Division of Intramural Research, National Institute of Allergy and Infectious Diseases, National Institutes of Health, Bethesda, MD USA; 3 Institute for Systems Biology, Seattle, WA USA

**Keywords:** Protein vaccines, Influenza virus, Influenza virus

## Abstract

Despite the importance of immunity against neuraminidase (NA), NA content and immunogenicity are neglected in current influenza vaccines. To address this, a recombinant N1/N2 NA vaccine (NAV) was developed. Stability assays were used to determine optimal temperature and buffer conditions for vaccine storage. The effect of divalent cation-related enhancement of NA stability and activity on N1 and N2 immunogenicity and efficacy against viral challenge was assessed. Differences in activity between N1 and N2 and cation-related activity enhancement did not translate into differences in immunogenicity or efficacy. NAV-vaccinated mice showed robust antibody titers against N1 and N2, and after challenge with influenza A (H1N1) virus, decreased viral titers and decreased antiviral and inflammatory responses by transcriptomic analysis. These findings provide guidance for optimal storage and assessment of NA-based vaccines and confirm the importance of NA in influenza vaccination strategies in attenuating viral replication and limiting inflammatory responses necessary to clear infection.

## Introduction

Influenza has precipitated four pandemics in the last century and endemic seasonal influenza continues to be responsible for significant morbidity and mortality worldwide. In the aftermath of the 1918 influenza pandemic, Smith et al. succeeded in isolating the first human influenza virus in 1933, thereby paving the way to work on development of a vaccine^1,2^. In 1945, Salk published the first trial evaluating an influenza vaccine, demonstrating an effectiveness of 58%^3^. Despite 75 years of remarkable scientific and technological advances since then, influenza vaccine effectiveness has not improved, with estimates in the twenty-first century ranging from as high as 60% to as low as 10%^4^. The focus of vaccine-induced immunity has generally been against influenza hemagglutinin (HA), the predominant influenza particle surface protein. However, numerous studies have implicated neuraminidase (NA) as an important correlate of protection^5,6^. In more recent studies, antibody titers against NA were shown to be a better predictor of clinical outcomes than antibody titers against HA^7^. Despite this, current influenza vaccines are only standardized with respect to HA content but not NA. Consequently, the NA content and immunogenicity of licensed influenza vaccines can vary tremendously, with some vaccines having nearly negligible amounts^8^. Differences in manufacturing process are likely to play a large role in NA content variability but stability of NA in vaccine preparations may be an additional important factor, especially in older vaccine lots. Homotetramer formation, an essential condition for NA enzymatic activity, is dependent on divalent cations to maintain stability^9,10^. Influenza vaccines are not typically formulated using buffers containing calcium or magnesium, which may lead to instability of the NA.

The field of vaccinology has experienced rapid evolution, providing for a potential renaissance in influenza vaccine development. For example, recombinant protein vaccines have been developed against multiple pathogens, including influenza^11^. Recombinant NA strategies may provide a simple but elegant solution for increasing NA content and immunogenicity, and could be given alone or in supplementation to standard HA-focused vaccines. Research into NA-based vaccines was pioneered by Johansson, Kilbourne and others during the late twentieth century but has made little progress in the new millennium. Purified NA vaccines (NAVs), and subsequently, recombinant NAVs have demonstrated robust immunogenicity and an ability to protect against lethal infectious challenge in animal models^12^. In addition, the protection afforded by NAV was shown to be broader than that of HA vaccines, displaying activity against heterologous strains^13^. Combination purified NAVs have also been developed which demonstrated that the inclusion of multiple NA types in a single vaccine did not impair the immune response induced by each type^14^. Furthermore, supplementation with purified NAV broadened the protection afforded by trivalent vaccine^15^. However, the storage conditions necessary to optimize NA recombinant protein stability and activity and the effects these have on immunogenicity have not been clearly determined^8,16^. In this study, we evaluated the effect of temperature and buffer on the NA activity of a dual recombinant protein NAV, and the subsequent impact of NA activity on efficacy and immunogenicity of the vaccine in mice.

## Results

### The impact of temperature and divalent-cation-containing buffer on the stability of N1 and N2

**Fig. 1:**
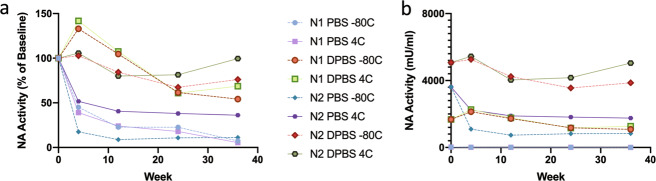
N1 and N2 NA activity over 36 weeks under different temperature and buffer conditions in vitro. NA activity of N1 and N2 stored in parallel at −80 °C and 4 °C, in PBS and DPBS (total eight samples), was measured using MU-NANA assay at weeks 0, 4, 12, 24, and 36. Activity presented as a percent (**a**) using week 0 as a baseline or as absolute activity (**b**). Points represent individual samples.

**Fig. 2:**
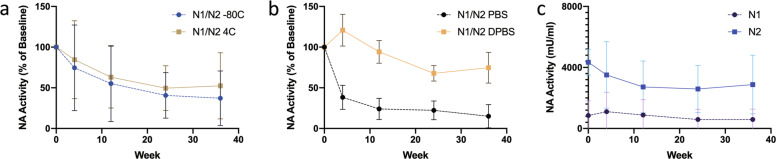
Grouped analysis demonstrating the effects of storage temperature, buffer choice, and NA type on NA activity in vitro over 36 weeks. NA activity measured by MU-NANA assay at weeks 0, 4, 12, 24, and 36 was grouped by temperature conditions (**a**), buffer choice (**b**), and NA type group (**c**). In **a**, N1/N2 −80 °C represents mean activity of N1 PBS −80 °C, N1 DPBS −80 °C, N2 PBS −80 °C, and N2 DPBS −80 °C and N1/N2 4 °C represents mean activity of N1 PBS 4 °C, N1 DPBS 4 °C, N2 PBS 4 °C, and N2 DPBS 4 °C. In **b**, N1/N2 PBS represents mean activity of N1 PBS −80 °C, N1 PBS 4 °C, N2 PBS −80 °C, and N2 PBS 4 °C and N1/N2 DPBS represents mean activity of N1 DPBS −80 °C, N1 DPBS 4 °C, N2 DPBS −80 °C, and DN2 PBS 4 °C. In **c**, N1 represents mean activity of N1 PBS −80 °C, N1 PBS 4 °C, N1 DPBS −80 °C, and N1 DPBS 4 °C and N2 represents mean activity of N2 PBS −80 °C, N2 PBS 4 °C, N2 DPBS −80 °C, and N2 DPBS 4 °C. Activity presented as a percent (**a**, **b**) using week 0 as a baseline or as absolute activity in mU/ml (**c**). Points represent means and error bars represent standard deviation.

The stability of recombinant N1 and N2 as measured by NA activity was tested under various storage conditions with emphasis on temperature (−80 °C vs 4 °C) and buffer solution [PBS (phosphate-buffered saline) vs divalent-cation-containing DPBS (Dulbecco’s PBS containing calcium and magnesium)]. NA activity of N1 in PBS or DPBS did not differ between samples stored at 4 °C and at −80 °C over the course of 36 weeks (Figs. 1a, b and 2a). The N2 in PBS sample appeared to maintain higher activity stored at 4 °C compared to −80 °C. The N2 in DPBS sample also appeared to maintain higher NA activity at 4 °C compared to −80 °C at weeks 24 and 36 (100% vs 76%). However, when averaged across N1 and N2 samples as well as PBS and DPBS buffer conditions, there were no statistically significant differences between freeze–thaw storage at −80 °C and storage at 4 °C (Fig. 2a and Supplemental Table 1). DPBS buffer was effective at preserving higher NA activity, compared to PBS, in both N1 and N2 over 36 weeks regardless of temperature (Fig. 2b and Supplemental Table 2). Mean activity as a percent of baseline for DPBS samples was higher than PBS at week 4 (120.8% vs 38.4%, *p* < 0.001), week 12 (94.2% vs 24.0%, *p* < 0.001), week 24 (67.8% vs 22.4%, *p* < 0.001), and week 36 (74.7% vs 15.0%, *p* = 0.002).

**Fig. 3:**
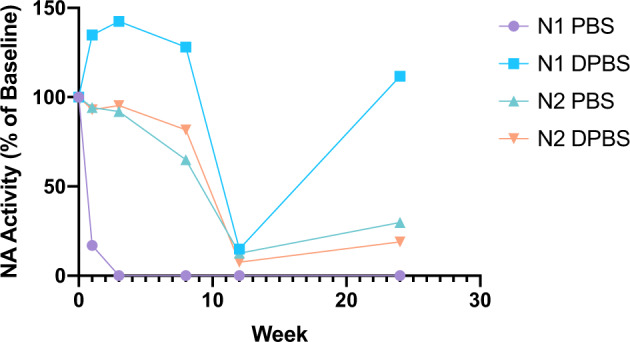
NA activity of N1 and N2 stored in PBS or DPBS at room temperature for 24 weeks. NA activity of N1 and N2 in PBS or DPBS at room temperature was measured using MU-NANA assay in triplicate at weeks 0, 3, 8, 12, and 24 using week 0 as a baseline. Points represent individual samples.

**Fig. 4:**
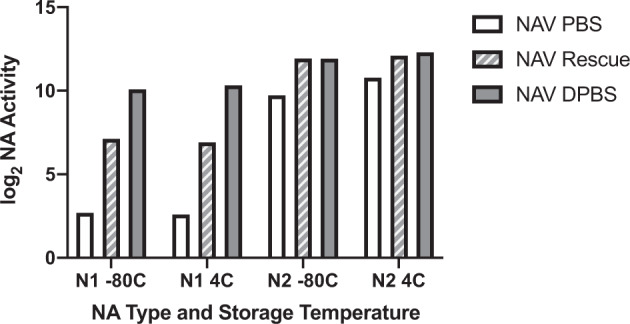
Calcium and magnesium partially rescued NA activity in vitro of N1 and N2 stored at −80 °C or 4 °C in PBS. N1 and N2 stored at −80 °C or 4 °C in PBS or DPBS for 36 weeks. PBS samples were rescued with DPBS and log_2_-transformed NA activities of NAV PBS, NAV rescue, and NAV DPBS samples were compared. Bars represent individual samples.

Mean N2 activity under all conditions was generally higher than N1, though this only reached statistical significance on day 0 (4346 mU/ml vs 851.4 mU/ml, *p* = 0.002) (Fig. 2c and Supplemental Table 3). Activity of N1 in PBS appeared to plummet by week 4 but was better maintained in DPBS. A drop in N1 activity was also apparent when stored in PBS at room temperature (RT) for 1 week and beyond (Fig. 3). Similarly, the N2 in PBS sample and the N1 and N2 in DPBS samples maintained their NA activity at RT for 8 weeks. NA activity could be increased by addition of calcium and magnesium to the 36-week-old sample of NA in PBS. While activity of N2 in PBS could be rescued to a similar level as displayed by N2 kept in DPBS for the entire 36 weeks, N1 in PBS only had a small increase in activity with addition of divalent cations (Fig. 4).

### NAV PBS and NAV DPBS induce robust but similar NA inhibition titers in mice

**Fig. 5:**
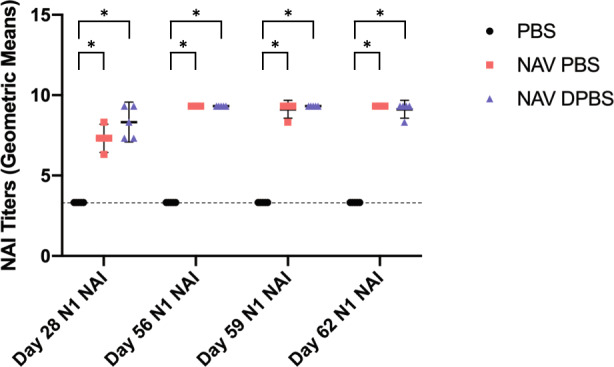
Immunogenicity of NAV PBS and NAV DPBS as assessed by NAI titers against N1. Mice were vaccinated with NAV in PBS (red squares), NAV in DPBS (blue triangles), and PBS control (black circles) on days 0 and 28 and challenged with 10^5^ TCID_50_ A/Bethesda/MM2/2009 (H1N1) on day 56. Blood was collected on days 28, 56, 59, and 62 for assessment of NAI titers against N1 (*n* = 5 mice per condition per time point except *n* = 10 on day 56). Points represent log_2_ geometric means of individual mice and error bars represent means and standard deviations. Dotted line represents minimal limit of detection. ANOVA and post-hoc Tukey test were performed where *p < 0.0001.

**Fig. 6:**
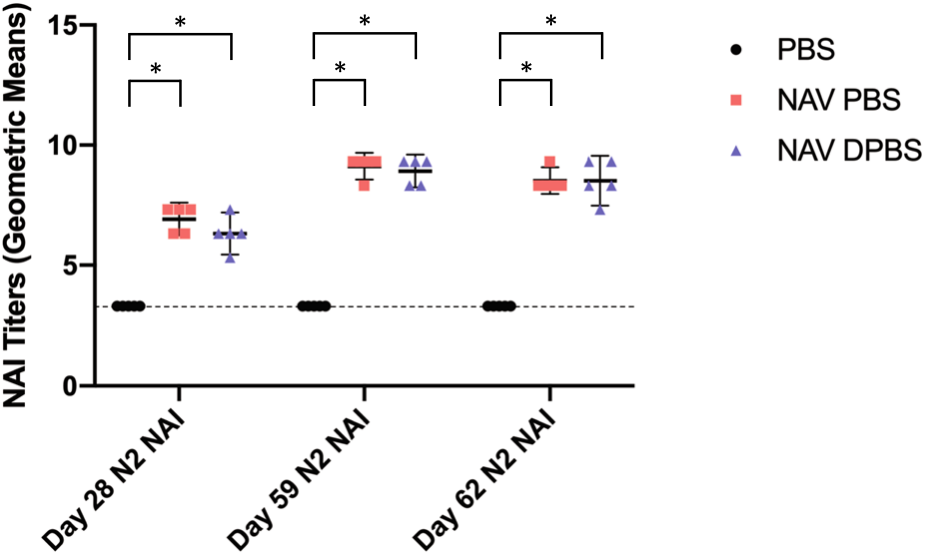
Immunogenicity of NAV PBS and NAV DPBS as assessed by NAI titers against N2. Mice were vaccinated with NAV in PBS (red squares), NAV in DPBS (blue triangles), and PBS control (black circles) on days 0 and 28 and challenged with 10^5^ TCID_50_ A/Bethesda/MM2/2009 (H1N1) on day 56. Blood was collected on days 28, 59, and 62 for assessment of NAI titers against N2 (*n* = 5 mice per condition per time point). Points represent log_2_ geometric means of individual mice and error bars represent means and standard deviations. Dotted line represents minimal limit of detection. ANOVA and post-hoc Tukey test were performed where *p < 0.0001.

Immunogenicity to NAV PBS and NAV DPBS stored at 4 °C was robust (Figs. 5 and 6). Twenty-eight days after the first dose of vaccine, serum NA inhibition (NAI) titers against N1 increased significantly in mice given NAV PBS (mean difference (MD) = 4.00, 95% CI 2.81–5.19, *p* < 0.0001) and NAV DPBS (MD = 5.00, 95% CI 3.81–6.19, *p* < 0.001) compared to the PBS control group (Supplemental Table 4). Titers were similar between mice given NAV PBS and those given NAV DPBS (MD = 1.00, 95% CI −0.19–2.19, *p* = 0.11). Similar findings were observed with NAI titers against N2, which were significantly higher in mice given NAV PBS (MD = 3.60, 95% CI 2.73–4.47, *p* < 0.0001) and NAV DPBS (MD = 3.00, 95% CI 2.13–3.87, *p* < 0.0001) but not different between vaccine groups (MD = −0.60, −1.47–0.27, *p* = 0.20) (Supplemental Table 5). NAI titers to N1 and N2 increased further after the second vaccine dose (in some cases above the maximum limit of detection on the assay) and persisted to be high through viral challenge in both vaccine groups. ANOVA could not be performed on day 56 to compare N1 NAI titers since all NAV PBS and NAV DPBS mice had titers that reached the maximum level of detection and there was no standard error. Subsequent ANOVA analyses for both N1 and N2 NAI titers on days 59 and 62 were all significant (*p* < 0.0001) with post-hoc analyses all determining NAV PBS and NAV DPBS mice had significantly higher titers than the PBS control group (all *p* < 0.0001) while the differences in titers between NAV PBS and NAV DPBS were not significant (Supplemental Tables 6–9). The control group had serum NAI titers against N1 and N2 below the assay limit of detection throughout the entire study, including viral challenge. Of note, insufficient sample was available to test NAI against N2 on day 56.

### NAV PBS and NAV DPBS provide similar protection against influenza challenge in mice

**Fig. 7:**
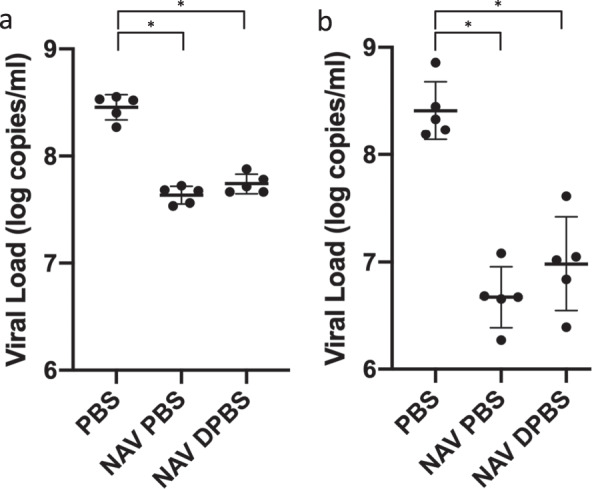
NAV reduced pulmonary viral loads in mice challenged with A/California/04/2009 (H1N1). Mice were vaccinated with NAV in PBS, NAV in DPBS, and PBS control on days 0 and 28 and challenged with 10^5^ TCID_50_ A/Bethesda/MM2/2009 (H1N1) on day 56. Pulmonary viral loads on day 3 (**a**) and day 6 (**b**) (*n* = 5 at each time point) after challenge were assessed by PCR. Points represent viral loads of individual mice and error bars represent means with standard deviations. ANOVA and post-hoc Tukey test were performed where *p < 0.0001.

To test the vaccine efficacy in the presence and absence of divalent cations, vaccinated mice were challenged with influenza on day 56 and viral titers were obtained (Fig. 7). Mice vaccinated with NAV PBS or NAV DPBS had lower viral loads (VLs) on day 59 (post-challenge day 3) and day 62 (post-challenge day 6) compared to control (PBS) vaccinated mice. Three days after vaccination, NAV PBS mice had −0.822 log copies/ml (95% CI, −0.989 to −0.655, *p* < 0.0001) compared to PBS mice while NAV DPBS mice had −0.716 log copies/ml (95% CI, −0.883 to −0.549, *p* < 0.0001) compared to PBS mice. Six days after vaccination, NAV PBS mice had −1.742 log copies/ml (95% CI, −2.316 to −1.168, *p* < 0.0001) compared to PBS mice while NAV DPBS mice had −1.430 log copies/ml (95% CI, −2.004 to −0.856, *p* < 0.0001) compared to PBS mice. VLs were not statistically different between mice vaccinated with NAV PBS and those vaccinated with NAV DPBS at either day 59 or day 62 (Supplemental Tables 10, 11).

### Post-influenza challenge inflammatory and antiviral transcriptomic responses are reduced in NAV PBS- and NAV DPBS-vaccinated mice

**Fig. 8:**
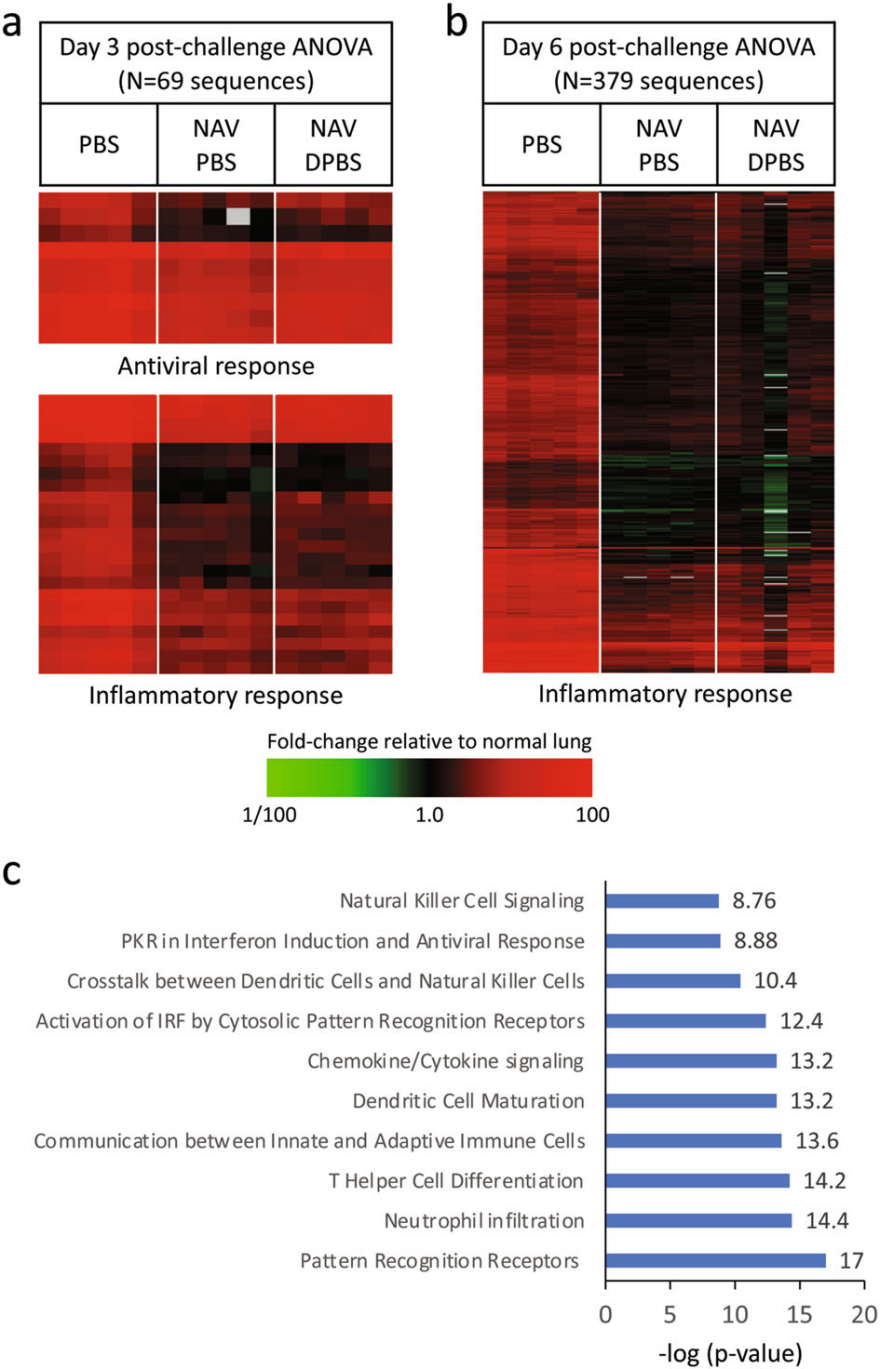
NAV PBS and NAV DPBS vaccination reduced antiviral and inflammatory response gene expression following viral challenge. Expression microarray analysis was performed on total RNA isolated from lungs on days 3 and 6 post-challenge (*n* = 5 mice per condition per time point). **a** Heatmaps showing expression of antiviral and inflammatory response genes identified by ANOVA (*p* < 0.001) that showed >2-fold change in expression between PBS, NAV PBS, and NAV DPBS vaccinated groups on day 3 post-viral challenge. Due to the small number of sequences (*n* = 69) identified at this timepoint, pathway classification analysis was not performed (gene IDs are shown in Supplemental Figs. 1 and 2). **b** Heatmap showing expression of antiviral/immune response genes identified by ANOVA (*p* < 0.001 with >2-fold change in expression) on day 6 post-viral challenge. Red indicates higher expression of sequences in infected mouse lung compared to normal unvaccinated, uninfected, age-matched mouse lung, while green indicates lower expression, and black indicates no change. **c** Pathway classification of genes identified by ANOVA on day 6 post-viral challenge.

To measure the effects of NAV PBS and NAV DPBS vaccination on the host gene expression response, cDNA expression microarray analysis was performed on total RNA isolated from whole lung on days 3 and 6 post-infection with 10^5^ TCID50 of 2009 H1N1pdm virus (*n* = 5 animals per condition per timepoint). A *t*-test comparing mock-infected and virus-infected animals identified 2341 and 3972 sequences with expression levels that changed significantly (at least two-fold, *p* value <0.001) on days 3 and 6 post-challenge, respectively, in response to viral infection. Analysis of Variance (ANOVA) was then used to identify virus-responsive genes with expression levels that differed (two-fold, *p* value <0.001) between PBS-, NAV PBS-, and NAV DPBS-vaccinated groups. This analysis identified 69 and 379 sequences on days 3 and 6 post-challenge, respectively. As shown in Fig. 8a and Supplemental Fig. 1, PBS-vaccinated mice showed significantly higher expression of antiviral response and inflammatory response genes compared to NAV PBS- and NAV DPBS-vaccinated groups on day 3 post-challenge. Similarly, on day 6 post-challenge, NAV PBS and NAV DPBS groups showed significantly less expression of immune/antiviral genes compared to PBS-vaccinated mice (Fig. 8b and Supplemental Fig. 2). Shown in Fig. 1c, pathway classification indicated NAV PBS and NAV DPBS vaccination resulted in a significant reduction in many antiviral responses, including pattern recognition, IRF activation, and interferon responses; inflammatory responses, including NK cell and neutrophil responses; and chemokine/cytokine signaling, lymphocyte, and dendritic cell responses. No significant differences were observed by *t*-test between NAV PBS and NAV DPBS vaccination at either day 3 or day 6 post-challenge.

## Discussion

The findings in this study are helpful in guiding storage practices for NA-based vaccines but also explore critical questions regarding the relationships between NA stability, activity, immunogenicity, and potential vaccine efficacy. Generally, NA is stored at 4 °C per manufacturer’s recommendations and investigator experience, but there is a paucity of published data available to support this practice^17,18^. The data from this study confirm these practices, demonstrating similar stability of activity for vaccine stored at 4 °C as compared to −80 °C. This is advantageous for NA-based vaccines, since storage at 4 °C is less resource intensive than storage at −80 °C. DPBS buffer helped to maintain activity for all samples at all temperatures and interestingly, seemed to diminish the destabilizing effect of the freeze–thaw process on N2. It is important to note, since the −80 °C samples underwent freeze–thawing at each time point, there may be an additional decrease in activity as a result of the freeze–thaw process itself rather than just storage at −80 °C. Surprisingly, the samples in DPBS were quite stable even at RT for at least 3 weeks, which is also helpful to know for vaccine storage, especially in low resource areas. However, RT storage could also increase the risk of microbial growth, which was not assessed in this study. Furthermore, the optimal temperature storage of HA, the chief antigen present in standard influenza vaccines, was not investigated in this study though these vaccines are also typically stored at 4 °C^19–23^. The RT stability experiment also showed an unexpected increase in activity of the N1 in DPBS sample above baseline from weeks 1 to 12. The reason for this is unclear, but the finding may indicate that divalent-cation-related enhancement of NA activity may not be an instantaneous process. It is also unclear why activity dropped precipitously for all samples at week 12 before rebounding to different degrees at week 24. The week 24 results were repeated the next day for N1 and N2 in DPBS (data not shown), showing similar results and confirming the NA activity was not spuriously elevated at week 24. However, since the samples were run longitudinally, the week 12 results were not able to be repeated to verify the observation of low NA activity. Factors that may affect multiple wells across different plates are limited but could be a result of suboptimal 4-methylumbelliferyl-*N*-acetylneuraminicacid (MU-NANA) reagent concentration at week 12.

N2 activity was generally higher when compared to N1 across all sets of conditions at all time points. Though this difference only reached statistical significance on day 0, the lack of significance at other time points was largely driven by high variability across samples under different conditions. There are limited data available in the literature comparing activity of NAs of different types that may shed light whether the observed difference is reflective of N1 and N2 in general, or recombinant N1 from A/California/04/2009 and recombinant N2 from A/Switzerland/9715293/2013 specifically. In a study from 1977, all N1-containing vaccines had consistently lower NA activity than the two H3N2 vaccines, with four out of six H1N1 vaccines having undetectable activity^24^. The N1 samples in our study also appeared to be highly vulnerable to loss of stability in the absence of calcium and magnesium. Again, it is not clear if this holds universally true for all N1 protein or if this is specific to this particular recombinant product from A/California/04/2009. The use of the vasodilator-stimulated phosphoprotein (VASP) tetramerization domain is an additional confounding factor since NA protein in typical split or subunit vaccines lacks this domain. Furthermore, the VASP tetramerization domain may potentially stabilize N1 and N2 to different degrees.

Knowing the importance of divalent cations for stability and hence, activity of NA, it is not surprising that DPBS buffer increased the activity of both N1 and N2 under all sets of conditions. It is interesting that for N1 samples at both −80 °C and 4 °C activity appeared to be higher at week 4 compared to baseline, also supporting that divalent-cation-related enhancement of NA activity, particularly in N1, may take a long time to reach maximum effect. Similarly, calcium and magnesium were able to increase the activity of N2 in PBS at 36 weeks to similar levels as N2 in DPBS, but N1 in PBS could only be increased partially, suggesting either that there was some permanent loss of stability of N1 in PBS or that enhancement of activity occurred more slowly in N1 compared to N2. An important limitation of the in vitro portion of this study was the lack of biological replicates which limited the ability to perform statistical comparisons. This was partially addressed through grouping of conditions which allowed for statistical comparisons but suffered from increased variability due to heterogeneity of conditions. This technique could not be performed to statistically support the observations of late divalent cation rescue on PBS samples or to more clearly characterize the stability of NA at RT.

Despite the differences in activity observed between N1 and N2, immunogenicity appeared to be fairly robust with N1 and N2 inducing similar NAI titers, questioning whether critical epitopes are actually lost in the setting of instability. This is in contrast to a previous study using purified NA which found modest improvement in the immunogenicity of calcium supplemented N1^25^. The presence of the VASP tetramerization domain in recombinant NA may confound the comparison with purified NA or standard vaccines, since the domain may provide sufficient stability to preserve epitopes needed for a successful humoral response even in the absence of divalent cations. Alternatively, it may be possible that in vivo extracellular calcium and magnesium concentrations are sufficient to rescue stability leading to comparable antibody titers in mice given PBS-buffered vaccine and DPBS-buffered vaccine. However, it is important to note that multiple samples reached the maximum level possible on the NAI assay which may limit comparisons. It is also regrettable that N2 titers were not available on day 56, though considering that N1 titers were generally at or near the maximum limit of detection between days 56, 59, and 62 and N2 titers were at comparable levels on days 59 and 62, it is unlikely day 56 N2 titers would have provided additional information. These findings also confirm that the high NAI titers on days 59 and 62 are not a response to viral challenge, but rather to the second dose of vaccine on day 28.

The comparable efficacy of PBS NAV and DPBS NAV in reducing the VL in vaccinated mice also supports the previous findings demonstrating a disconnect between vaccine NA activity and immunogenicity. The higher NA activity of the N1 component in NAV DPBS compared to NAV PBS did not result in any further reduction in VL after H1N1 challenge. It has been previously shown that while distinct NA types induce NAI titers to different degrees, this variance cannot be accounted for by the difference in NA activity^17^. In contrast, since NA amount correlates well with activity and immunogenicity, NA activity can be used to predict immunogenicity only when comparing samples containing the same NA type^17,26^. The observed VL reduction correlates with that seen in other studies of NA-based vaccines, though it was not quite as robust as the 4 log reduction observed in N2-immunized mice challenged with homotypic and even heterotypic H3N2 virus^16^.

However, this study, which used a combination of N1 and N2 vaccine, demonstrated a significant effect on the overall disease response as indicated by transcriptomic analysis. The significant reduction in expression of antiviral and inflammatory responses in vaccinated mice post-challenge suggests that the pathogenesis of influenza infection was greatly muted. Compared to control animals, NAV-vaccinated animals demonstrated a superior reduction of VLs and decreased antiviral and inflammatory responses, further confirming the observed positive effects of anti-NA immunity on clinical outcomes demonstrated in human challenge trials^7^. Mechanistically, these findings suggest anti-NA immunity effectively impairs influenza pathogenesis and replication, necessitating decreased immune activation to clear infection, leading to decreased symptoms secondary to inflammatory responses.

Although the inclusion of calcium and magnesium in vaccine buffer did not have a significant effect on immunogenicity or efficacy of the vaccine, there may still be advantages to the use of divalent-cation-containing buffer for NA-based vaccines, including a positive effect on maintaining stability over the long term. While this study used NAV that was freshly formulated with DPBS to immunize mice, this hypothesis can be readily tested by comparing efficacy and immunogenicity of PBS NAV and DPBS NAV after long-term storage. The effect of DPBS on HA should also be explored before it can be used in combination HA+NA vaccines, including as an improvement to standard vaccines already on the market.

The results from this study confirm that this NA recombinant vaccine strategy, NAV, has potential as a standalone vaccine. Further work, comparing its efficacy to approved quadrivalent vaccine or using NAV as a supplement to approved influenza vaccines where it can complement HA-based immunity, will be essential. In this case, the combination of recombinant N1 and N2 in a single vaccine did not impair robust induction of anti-NA antibodies or prevent protection against H1N1 challenge, as demonstrated by a reduction in VL and decreased inflammation. This strategy could also be scaled to a 9-valent vaccine covering all nine NA subtypes, with potential as a universal vaccine. NAV was highly stable and even in the absence of a divalent-cation-containing buffer was immunogenic and protective. Studies in both animals and humans suggest that NA immunity can play a significant role in reduction of clinical illness and offer increased breadth of protection^5,7,8,16^. In addition, a recombinant NAV could be updated to better represent currently circulating NA strains when necessary. Evaluating this vaccine in humans will allow us to determine if these promising observations of immunogenicity and efficacy translate. Evaluation of this vaccine as an independent or combination strategy with current inactivated influenza vaccine is critical as it may offer a significant improvement in vaccine performance while more broadly protective, “universal” vaccines are developed.

## Methods

### Recombinant protein production

N1 from A/California/04/2009 (H1N1) and N2 from A/Switzerland/9715293/2013 (H3N2) with the addition of a VASP tetramerization domain were designed as previously described^27^ with a Strep-tag II for purification instead of the hexahistidine tag used in the reference study. Both N1 and N2 proteins were produced in Spodoptera frugiperda (Sf9) insect cells using the Bac-to-Bac baculovirus expression system (Thermo Fisher Scientific, Waltham, MA) and purified using Strep-Tactin Sepharose (IBA GmbH, Gottingen, Germany) as previously described^28^. A Bicinchoninic Acid Protein Assay Kit (Thermo Fisher Scientific, Waltham, MA) was used for protein quantification.

### Storage conditions and timepoints

N1 and N2 were each diluted either in PBS (Quality Biological, Gaithersburg, MD) or DPBS (Thermo Fisher Scientific, Waltham, MA). The recipe for PBS, as provided by the manufacturer, is sodium phosphate dibasic (795 mg/l, 5.6 mM), potassium phosphate monobasic (1.06 mM), and sodium chloride (9000 mg/l, 154 mM). The recipe for DPBS, as provided by the manufacturer, is calcium chloride (100 mg/l, 0.9 mM), magnesium chloride (100 mg/l, 0.49 mM), potassium chloride (200 mg/l, 2.66 mM), potassium phosphate monobasic (200 mg/l, 1.47 mM), sodium chloride (8000 mg/l, 137.93 mM), and sodium phosphate dibasic (2160 mg/l, 8.06 mM). Samples were stored at −80 °C and at 4 °C for a total of eight different sets of combinations of buffer and temperature conditions. NA stored at −80 °C was thawed at each timepoint and then returned to frozen storage until the next timepoint. NA activity was tested at weeks 0, 4, 12, 24, and 36. NA in PBS at week 36 was subsequently “rescued” through dilution in DPBS to compare its activity to NA in PBS at 36 weeks and NA in DPBS at 36 weeks. The stability experiment was also repeated with N1 in PBS, N1 in DPBS, N2 in PBS, and N2 in DPBS at RT and activity was tested at weeks 0, 3, 8, 12, and 24.

### NA activity measurement

N1 and N2 activity was measured using MU-NANA (MilliporeSigma, St. Louis, MO) as substrate, adapted from the method used by Sultana et al.^17^. In brief, N1 and N2 were initially diluted to working concentration in either PBS or DPBS. Samples were prepared in a black 96-well plate. Two-fold dilutions using PBS of *Vibrio cholerae* NA (MilliporeSigma, St. Louis, MO) were performed in duplicate and used as a positive control to create a standard curve. Two-fold dilutions, using either PBS or DPBS, were performed in triplicate on each sample. An equal amount of 20 µM MU-NANA was added to each well. The plate was incubated at 37 °C for 1 h. The reaction was stopped by the addition of 100 µl of 0.1 M glycine, pH 10.7, 20% EtOH. Fluorescence was measured by a Synergy HT plate reader (BioTek, Winooski, VT), with excitation at 360 nm and emission at 460 nm. Data were compiled in PRISM 8 version 8.3.0 (GraphPad Software, San Diego, CA). Absolute NA activity was extrapolated using the first dilution that had fluorescence readings within the linear portion of the standard curve across an entire group. NA activity (as a comparison to baseline) was also calculated by comparing and averaging optical density (OD) values at each dilution to baseline OD values. NA activity was also grouped by temperature condition, buffer choice, and NA type at each timepoint for comparison, with standard deviations calculated using PRISM 8.

### Vaccination and viral challenge of mice

N1 and N2 were produced approximately 4 and 53 months prior to vaccination and stored at −80 °C until the day of vaccination. On the day of vaccination, NAV was diluted either in PBS (NAV PBS) or DPBS (NAV DPBS) as buffer solutions to target concentrations of 100 µg/ml of N1 and 100 µg/ml of N2. NAV was stored at 4 °C in between administration of the first and second doses of vaccine. The same lot was used for first and second doses in each group. BALB/c mice (8-week-old female BALB/c mice, Jackson Laboratories, Bar Harbor, ME) were vaccinated intramuscularly (IM) with NAV DPBS (*n* = 10), NAV PBS (*n* = 10), or PBS control (*n* = 10). Vaccination occurred on day 0 with 10 µg of each NA and on day 28 with 1 µg of each NA in the two vaccine groups. Mice were bled 200 µl on day 28 and day 56 to collect serum for assessment of immunogenicity. Mice were challenged intranasally with 10^5^ TCID_50_ A/Bethesda/MM2/2009 (H1N1) on day 56 and observed daily for adverse effects. Of note, A/Bethesda/MM2/2009 (H1N1) was derived via reverse genetics from A/California/04/2009 (H1N1) and differs by three amino acid changes, one in the HA (A388V) and two in the NA (V106I and N248D). Mice in each group were euthanized in equal numbers on day 59 and day 62 for assessment of VL and transcriptomic analysis of lung tissue. Serum was collected from sacrificed mice. All experimental animal work was performed in accordance with United States Public Health Service Policy on Humane Care and Use of Laboratory in Animals at the National Institute of Allergy and Infectious Diseases (NIAID) of the National Institutes of Health (NIH) following approval of the animal safety protocols by the NIAID Animal Care and Use Committee.

### Assessment of immunogenicity

Immunogenicity of the vaccine was assessed through the use of a standard NAI assay on serum collected from vaccinated mice. Titers for N1 NAI were generated using an H6N1 assay virus with an identical N1 to the vaccine N1 and titers for N2 NAI were generated using an H6N2 assay virus with an identical N2 to the vaccine N2, as per previously reported standard methods^29,30^. All measurements were made in triplicate. Raw NAI titers were transformed into geometric means using log_2_. Immunogenicity was tested on days 28, 56, 59, and 62. Data were compiled and standard deviations were calculated using PRISM 8.

### Assessment of VL

Lungs were obtained from euthanized mice on days 59 and 62 and homogenized. After RNA extraction, VLs were measured using a one-step real-time quantitative reverse transcription-PCR (RT-PCR) for the influenza A virus matrix 1^31^. A standard curve with an external standard was used to calculate copy number as previously described^32^. Data were compiled and 95% confidence intervals were calculated using PRISM 8.

### RNA isolation and expression microarray analysis

Lungs from euthanized mice were collected and homogenized in Trizol, total RNA was isolated following manufacturer’s protocol (Thermo Fisher Scientific, Waltham, MA) and purified using the RNeasy Mini Kit (Cat #74106). Gene expression profiling experiments were performed using Agilent Mouse Whole Genome 44K microarrays. Fluorescent probes were prepared using Agilent QuickAmp Labeling Kit according to the manufacturer’s instructions. Each RNA sample was labeled and hybridized to individual arrays. Spot quantitation was performed using Agilent’s Feature Extractor software. The complete MIAME-compliant^33^ microarray dataset has been deposited in NCBI’s Gene Expression Omnibus^34^.

### Statistical analyses

PRISM 8 was used to make comparisons between NA activity of grouped samples with respect to storage temperature, buffer choice, and NA type. Two-tailed *t*-tests were performed to compare groups at each timepoint, with the two-stage step-up method of Benjamini et al. used to correct for multiplicity^35^. PRISM 8 was used to perform ANOVA on NAI titers to identify presence of differences between groups vaccinated with PBS, NAV PBS, and NAV DPBS at each time point for both N1 and N2. Tukey’s post-hoc test was used to perform comparisons on pairs of groups. ANOVA and Tukey testing were also performed to compare VL titers between groups on days 3 and 6 post-challenge. Statistical analysis of transcriptomic data was performed using Genedata Analyst 9.0 (Genedata, Basel, Switzerland). Data normalization was performed using central tendency followed by relative normalization using pooled RNA from mock infected mouse lung (*n* = 4) as a reference. Transcripts showing differential expression (two-fold, *p* < 0.001) between infected and control animals were identified by standard *t*-test. ANOVA was used to identify transcripts showing differential expression (two-fold, *p* value <0.001) between PBS-, NAV PBS-, and NAV DPBS-vaccinated groups. The Benjamini–Hochberg procedure was used to correct for false-positive rate in multiple comparisons. Ingenuity Pathway Analysis (IPA) was used for gene ontology and pathway classification.

### Reporting summary

Further information on research design is available in the Nature Research Reporting Summary linked to this article.

## Supplementary information

Supplementary Information

Reporting Summary

## Data Availability

The data that support the findings of this study are available from the corresponding author upon reasonable request. The transcriptomic data have been deposited in NCBI’s Gene Expression Omnibus with GEO Series accession number GSE160010.
