# Supplementary material for: The effect of calcium and magnesium on activity, immunogenicity, and efficacy of a recombinant N1/N2 neuraminidase vaccine
Source: NPJ Vaccines. 2021 Apr 6;6:48. doi: 10.1038/s41541-021-00310-x (PMC8024250; doi:10.1038/s41541-021-00310-x)
Supplement: Supplementary file 1 — Supplementary Information [file 41541_2021_310_MOESM1_ESM.pdf]

Supplementary Table 1: Multiple T-test comparison of NA activity of temperature grouped samples

Week	P value	Mean of N1/N2 -80C	Mean of N1/N2 4C	Difference	SE of difference	Degrees of freedom
0		100.0	100.0	0.000	0.000	
4	0.788734	74.60	84.57	-9.969	35.58	6
12	0.802089	55.17	63.03	-7.865	30.02	6
24	0.665619	40.64	49.58	-8.938	19.68	6
36	0.584723	37.22	52.44	-15.22	26.36	6

Supplementary Table 2: Multiple T-test comparison of NA activity of buffer grouped samples

Week	P value	Mean of N1/N2 -80C	Mean of N1/N2 4C	Difference	SE of difference	Degrees of freedom
0		100.0	100.0	0.000	0.000	
4	0.000523	38.37	120.8	-82.43	12.24	6
12	0.000323	24.02	94.18	-70.17	9.536	6
24	0.000892	22.37	67.84	-45.48	7.468	6
36	0.002388	15.01	74.65	-59.64	11.86	6

Supplementary Table 3: Multiple T-test comparison of NA activity of NA-type grouped samples

Week	P value	Mean of N1/N2 -80C	Mean of N1/N2 4C	Difference	SE of difference	Degrees of freedom
0	0.001546	851.4	4346	-3494	637.8	6
4	0.105074	1107	3510	-2403	1260	6
12	0.110989	880.6	2724	-1843	986.7	6
24	0.054922	590.6	2592	-2001	841.6	6
36	0.066143	591.4	2876	-2284	1019	6

Supplementary Table 4: Tukey multiple comparisons test of day 28 N1 NAI titers

Tukey's multiple comparisons test	Mean 1	Mean 2	Mean Diff.	95.00% CI of diff.	Degrees of freedom	Summary	Adjusted P Value
PBS vs. NAV PBS	3.322	7.322	-4.000	-5.193 to -2.807	12	****	<0.0001
PBS vs. NAV DPBS	3.322	8.322	-5.000	-6.193 to -3.807	12	****	<0.0001
NAV PBS vs. NAV DPBS	7.322	8.322	-1.000	-2.193 to 0.1931	12	ns	0.1051

Supplementary Table 5: Tukey multiple comparisons test of day 28 N2 NAI titers

Tukey's multiple comparisons test	Mean 1	Mean 2	Mean Diff.	95.00% CI of diff.	Degrees of freedom	Summary	Adjusted P Value
PBS vs. NAV PBS	3.322	6.922	-3.600	-4.471 to -2.729	12	****	<0.0001
PBS vs. NAV DPBS	3.322	6.322	-3.000	-3.871 to -2.129	12	****	<0.0001
NAV PBS vs. NAV DPBS	6.922	6.322	0.6000	-0.2713 to 1.471	12	ns	0.1995

Supplementary Table 6: Tukey multiple comparisons test of day 59 N1 NAI titers

Tukey's multiple comparisons test	Mean 1	Mean 2	Mean Diff.	95.00% CI of diff.	Degrees of freedom	Summary	Adjusted P Value
PBS vs. NAV PBS	3.322	9.122	-5.800	-6.236 to -5.364	12	****	<0.0001
PBS vs. NAV DPBS	3.322	9.322	-6.000	-6.436 to -5.564	12	****	<0.0001
NAV PBS vs. NAV DPBS	9.122	9.322	-0.2000	-0.6357 to 0.2357	12	ns	0.4619

Supplementary Table 7: Tukey multiple comparisons test of day 59 N2 NAI titers

Tukey's multiple comparisons test	Mean 1	Mean 2	Mean Diff.	95.00% CI of diff.	Degrees of freedom	Summary	Adjusted P Value
PBS vs. NAV PBS	3.322	9.122	-5.800	-6.489 to -5.111	12	****	<0.0001
PBS vs. NAV DPBS	3.322	8.922	-5.600	-6.289 to -4.911	12	****	<0.0001
NAV PBS vs. NAV DPBS	9.122	8.922	0.2000	-0.4888 to 0.8888	12	ns	0.7250

Supplementary Table 8: Tukey multiple comparisons test of day 62 N1 NAI titers

Tukey's multiple comparisons test	Mean 1	Mean 2	Mean Diff.	95.00% CI of diff.	Degrees of freedom	Summary	Adjusted P Value
PBS vs. NAV PBS	3.322	9.322	-6.000	-6.436 to -5.564	12	****	<0.0001
PBS vs. NAV DPBS	3.322	9.122	-5.800	-6.236 to -5.364	12	****	<0.0001
NAV PBS vs. NAV DPBS	9.322	9.122	0.2000	-0.2357 to 0.6357	12	ns	0.4619

Supplementary Table 9: Tukey multiple comparisons test of day 62 N2 NAI titers

Tukey's multiple comparisons test	Mean 1	Mean 2	Mean Diff.	95.00% CI of diff.	Degrees of freedom	Summary	Adjusted P Value
PBS vs. NAV PBS	3.322	6.922	-3.600	-4.471 to -2.729	12	****	<0.0001
PBS vs. NAV DPBS	3.322	6.322	-3.000	-3.871 to -2.129	12	****	<0.0001
NAV PBS vs. NAV DPBS	6.922	6.322	0.6000	-0.2713 to 1.471	12	ns	0.1995

Supplementary Table 10: Tukey multiple comparisons test of post-challenge day 3 viral loads

Tukey's multiple comparisons test	Mean 1	Mean 2	Mean Diff.	95.00% CI of diff.	Degrees of freedom	Summary	Adjusted P Value
PBS vs. NAV PBS	8.454	7.632	0.8220	0.6546 to 0.9894	12	****	<0.0001
PBS vs. NAV DPBS	8.454	7.738	0.7160	0.5486 to 0.8834	12	****	<0.0001
NAV PBS vs. NAV DPBS	7.632	7.738	-0.1060	-0.2734 to 0.06138	12	ns	0.2488

Supplementary Table 11: Tukey multiple comparisons test of post-challenge day 6 viral loads

Tukey's multiple comparisons test	Mean 1	Mean 2	Mean Diff.	95.00% CI of diff.	Degrees of freedom	Summary	Adjusted P Value
PBS vs. NAV PBS	8.412	6.670	1.742	1.168 to 2.316	12	****	<0.0001
PBS vs. NAV DPBS	8.412	6.982	1.430	0.8555 to 2.004	12	****	<0.0001
NAV PBS vs. NAV DPBS	6.670	6.982	-0.3120	-0.8865 to 0.2625	12	ns	0.3484

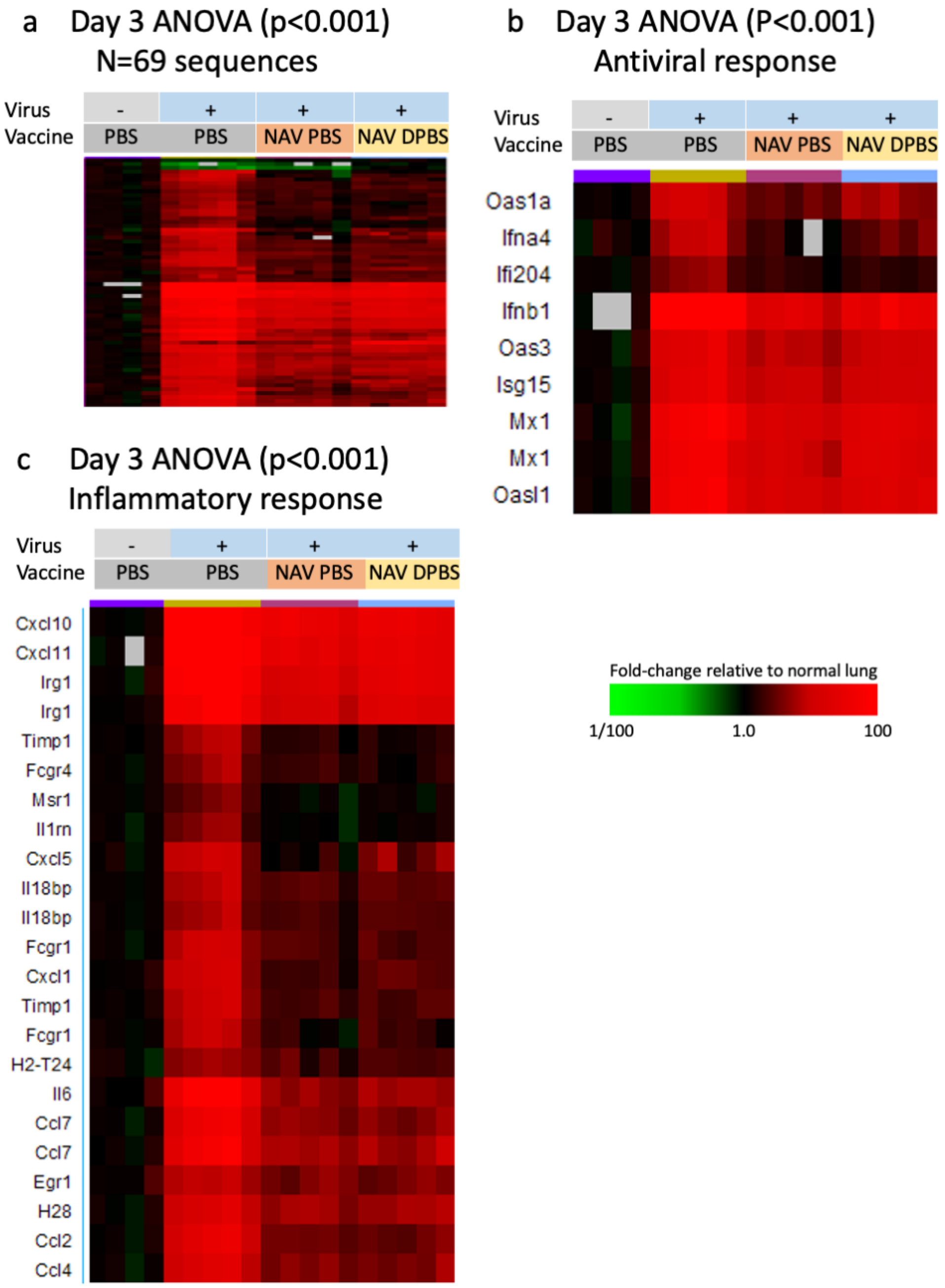

Supplementary Figure 1: Effect of NAV PBS and NAV DPBS vaccination on lung gene expression response on day 3 post-viral challenge. Expression microarray analysis was performed on total RNA isolated from lungs at day 3 post-challenge (n=5 mice per group). Heatmaps showing expression of antiviral and inflammatory response genes identified by ANOVA (p<0.001) that showed >2-fold change in expression between PBS, NAV PBS, and NAV DPBS vaccinated groups on day 3 post-viral challenge. (a) All sequences identified by ANOVA on day 3 post-challenge (n=69), (b) antiviral response genes and (c) inflammatory response genes. Sequences shown in red indicate higher expression in infected mouse lung compared to normal unvaccinated, uninfected, age-matched mouse lung; while green indicates lower expression, and black indicates no change.

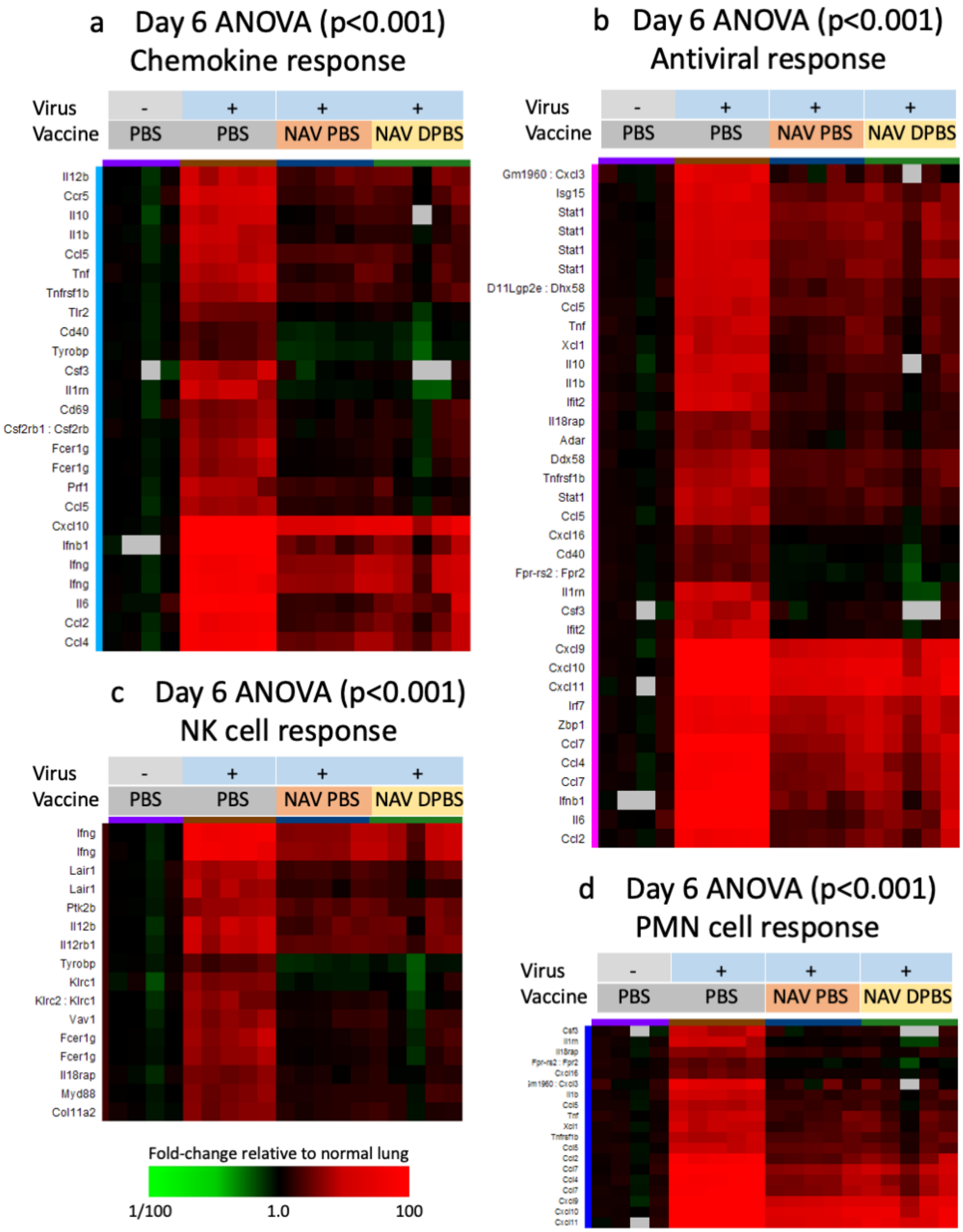

Supplementary Figure 2: Effect of NAV PBS and NAV DPBS vaccination on lung gene expression response on day 6 post-viral challenge. Expression microarray analysis was performed on total RNA isolated from lungs at day 6 post-challenge (n=5 mice per group). Heatmaps showing expression of select genes identified by ANOVA ($p<0.001$) that showed >2-fold change in expression between PBS, NAV PBS, and NAV DPBS vaccinated groups. (a)

chemokines response genes, (b) antiviral response genes, (c) natural killer (NK) cell related genes and (d) peritoneal mononuclear (PMN) cell related genes. Sequences shown in red indicate higher expression in infected mouse lung compared to normal unvaccinated, uninfected, age-matched mouse lung; while green indicates lower expression, and black indicates no change.
