# Supplementary material for: The effect of calcium and magnesium on activity, immunogenicity, and efficacy of a recombinant N1/N2 neuraminidase vaccine
Source: NPJ Vaccines. 2021 Apr 6;6:48. doi: 10.1038/s41541-021-00310-x (PMC8024250; doi:10.1038/s41541-021-00310-x)
Supplement: Supplementary file 2 — Reporting Summary [file 41541_2021_310_MOESM2_ESM.pdf]

nature research

Corresponding author(s): Giurgea, Luca

Last updated by author(s): Mar 1, 2021

# Reporting Summary

Nature Research wishes to improve the reproducibility of the work that we publish. This form provides structure for consistency and transparency in reporting. For further information on Nature Research policies, see our Editorial Policies and the Editorial Policy Checklist.

## Statistics

For all statistical analyses, confirm that the following items are present in the figure legend, table legend, main text, or Methods section.

| n/a | Confirmed | |
|---|---|---|
| ☐ | ☒ | The exact sample size (*n*) for each experimental group/condition, given as a discrete number and unit of measurement |
| ☐ | ☒ | A statement on whether measurements were taken from distinct samples or whether the same sample was measured repeatedly |
| ☐ | ☒ | The statistical test(s) used AND whether they are one- or two-sided<br>*Only common tests should be described solely by name; describe more complex techniques in the Methods section.* |
| ☒ | ☐ | A description of all covariates tested |
| ☐ | ☒ | A description of any assumptions or corrections, such as tests of normality and adjustment for multiple comparisons |
| ☐ | ☒ | A full description of the statistical parameters including central tendency (e.g. means) or other basic estimates (e.g. regression coefficient) AND variation (e.g. standard deviation) or associated estimates of uncertainty (e.g. confidence intervals) |
| ☐ | ☒ | For null hypothesis testing, the test statistic (e.g. *F*, *t*, *r*) with confidence intervals, effect sizes, degrees of freedom and *P* value noted<br>*Give P values as exact values whenever suitable.* |
| ☒ | ☐ | For Bayesian analysis, information on the choice of priors and Markov chain Monte Carlo settings |
| ☒ | ☐ | For hierarchical and complex designs, identification of the appropriate level for tests and full reporting of outcomes |
| ☒ | ☐ | Estimates of effect sizes (e.g. Cohen's *d*, Pearson's *r*), indicating how they were calculated |

*Our web collection on statistics for biologists contains articles on many of the points above.*

## Software and code

Policy information about availability of computer code

| | |
|---|---|
| Data collection | Data were collected in Excel for Mac 16.45. |
| Data analysis | Data were analyzed using PRISM 8 for macOS 8.3.0 with exception of the transcriptomic analysis which was performed using Genedata Analyst 9.0. |

For manuscripts utilizing custom algorithms or software that are central to the research but not yet described in published literature, software must be made available to editors and reviewers. We strongly encourage code deposition in a community repository (e.g. GitHub). See the Nature Research guidelines for submitting code & software for further information.

## Data

Policy information about availability of data

All manuscripts must include a data availability statement. This statement should provide the following information, where applicable:

- Accession codes, unique identifiers, or web links for publicly available datasets
- A list of figures that have associated raw data
- A description of any restrictions on data availability

The datasets generated during and/or analysed during the current study are available from the corresponding author on reasonable request. The transcriptomic data have been deposited in NCBI's Gene Expression Omnibus with GEO Series accession number GSE160010 (https://www.ncbi.nlm.nih.gov/geo/query/acc.cgi?acc=GSE160010)

# Field-specific reporting

Please select the one below that is the best fit for your research. If you are not sure, read the appropriate sections before making your selection.

☒ Life sciences ☐ Behavioural & social sciences ☐ Ecological, evolutionary & environmental sciences

For a reference copy of the document with all sections, see nature.com/documents/nr-reporting-summary-flat.pdf

# Life sciences study design

All studies must disclose on these points even when the disclosure is negative.

| | |
|---|---|
| Sample size | Sample sizes of 5 mice per group were used for analysis of immunogenicity and vaccine efficacy. Effect size was unknown for this novel vaccine candidate. The effect size of buffer choice was also unknown. Hence, a sample size of 5 was used to balance ethical and scientific aims. These sample sizes were sufficient to demonstrate statistical differences in immunogenicity, outcomes after viral challenge, and transcriptomics of antiviral and inflammatory responses following viral challenge between vaccinated mice and control group mice. |
| Data exclusions | No data were excluded |
| Replication | NA activity assays were tested in triplicate. NAI assays were tested in triplicate. PCR was performed in duplicate. Replicate values were averaged. |
| Randomization | Animals were assigned to experimental groups randomly. |
| Blinding | Animal data was collected in a blinded manner. |

# Reporting for specific materials, systems and methods

We require information from authors about some types of materials, experimental systems and methods used in many studies. Here, indicate whether each material, system or method listed is relevant to your study. If you are not sure if a list item applies to your research, read the appropriate section before selecting a response.

## Materials & experimental systems

| n/a | Involved in the study | |
|---|---|---|
| ☒ | ☐ | Antibodies |
| ☒ | ☐ | Eukaryotic cell lines |
| ☒ | ☐ | Palaeontology and archaeology |
| ☐ | ☒ | Animals and other organisms |
| ☒ | ☐ | Human research participants |
| ☒ | ☐ | Clinical data |
| ☒ | ☐ | Dual use research of concern |

## Methods

| n/a | Involved in the study | |
|---|---|---|
| ☒ | ☐ | ChIP-seq |
| ☒ | ☐ | Flow cytometry |
| ☒ | ☐ | MRI-based neuroimaging |

## Animals and other organisms

Policy information about studies involving animals; ARRIVE guidelines recommended for reporting animal research

| | |
|---|---|
| Laboratory animals | BALB/c mice were obtained from Jackson Laboratories (Bar Harbor, ME). Mice were 8-week-old females. |
| Wild animals | Study did not use wild animals. |
| Field-collected samples | Study did not use field-collected samples. |
| Ethics oversight | All experimental animal work was performed in accordance with United States Public Health Service Policy on Humane Care and Use of Laboratory in Animals at the National Institute of Allergy and Infectious Diseases (NIAID) of the National Institutes of Health (NIH) following approval of the animal safety protocols by the NIAID Animal Care and Use Committee. |

Note that full information on the approval of the study protocol must also be provided in the manuscript.
